# In Silico and In Vitro Mapping of Receptor-Type Protein Tyrosine Phosphatase Receptor Type D in Health and Disease: Implications for Asprosin Signalling in Endometrial Cancer and Neuroblastoma

**DOI:** 10.3390/cancers16030582

**Published:** 2024-01-30

**Authors:** Sophie Orton, Rebecca Karkia, Denis Mustafov, Seley Gharanei, Maria Braoudaki, Alice Filipe, Suzana Panfilov, Sayeh Saravi, Nabeel Khan, Ioannis Kyrou, Emmanouil Karteris, Jayanta Chatterjee, Harpal S. Randeva

**Affiliations:** 1 Warwick Medical School, University of Warwick, Coventry CV4 7AL, UK; sophie.n.orton@warwick.ac.uk (S.O.); s.gharanei@warwick.ac.uk (S.G.); kyrouj@gmail.com (I.K.); 2 College of Health, Medicine and Life Sciences, Brunel University London, Uxbridge UB8 3PH, UK; rebecca.karkia@nhs.net (R.K.); dm18abn@herts.ac.uk (D.M.); alice.filipe@brunel.ac.uk (A.F.); suzana.panfilov@brunel.ac.uk (S.P.); sayeh.saravi@brunel.ac.uk (S.S.); docnabeel31@gmail.com (N.K.); emmanouil.karteris@brunel.ac.uk (E.K.); 3 School of Life and Medical Sciences, University of Hertfordshire, Hatfield AL10 9JA, UK; m.braoudaki@herts.ac.uk; 4 Warwickshire Institute for the Study of Diabetes, Endocrinology and Metabolism (WISDEM), University Hospitals Coventry and Warwickshire NHS Trust, Coventry CV2 2DX, UK; 5 Aston Medical School, College of Health and Life Sciences, Aston University, Birmingham B4 7ET, UK; 6 Centre for Sport, Exercise and Life Sciences, Research Institute for Health & Wellbeing, Coventry University, Coventry CV1 5FB, UK; 7 College of Health, Psychology and Social Care, University of Derby, Derby DE22 1GB, UK; 8 Laboratory of Dietetics and Quality of Life, School of Food and Nutritional Sciences, Agricultural University of Athens, 11855 Athens, Greece; 9 Academic Department of Gynaecological Oncology, Royal Surrey NHS Foundation Trust Hospital, Guildford GU2 7XX, UK

**Keywords:** protein tyrosine phosphatase receptor type D, PTPRD, asprosin, endometrial cancer, glioblastoma, placenta

## Abstract

**Simple Summary:**

Protein Tyrosine Phosphatase Receptor Type D (PTPRD) plays a role in cell proliferation, differentiation, oncogenic transformation, and brain development and serves as an orexigenic asprosin receptor. This study investigates the expression of PTPRD in endometrial cancer (EC) and the placenta, as well as in glioblastoma (GBM). PTPRD is significantly upregulated at the mRNA and protein levels in patients with EC and GBM compared to healthy controls. In patients with EC, PTPRD is significantly downregulated by obesity. Using a tissue microarray, abundant PTPRD expression in low- and high-grade EC tumours is noted. Using liquid biopsies from EC patients, we show the expression of PTPRD in peripheral leukocytes. Moreover, asprosin treatment upregulates the expression of PTPRD in syncytialised placental cells in vitro, but not in EC cell lines. Collectively, our data suggest that PTPRD may have potential as a biomarker for malignancies such as EC and GBM, further implicating asprosin as a potential metabolic regulator in these cancers.

**Abstract:**

Background: Protein Tyrosine Phosphatase Receptor Type D (PTPRD) is involved in the regulation of cell growth, differentiation, and oncogenic transformation, as well as in brain development. PTPRD also mediates the effects of asprosin, which is a glucogenic hormone/adipokine derived following the cleavage of the C-terminal of fibrillin 1. Since the asprosin circulating levels are elevated in certain cancers, research is now focused on the potential role of this adipokine and its receptors in cancer. As such, in this study, we investigated the expression of PTPRD in endometrial cancer (EC) and the placenta, as well as in glioblastoma (GBM). Methods: An array of in silico tools, in vitro models, tissue microarrays (TMAs), and liquid biopsies were employed to determine the gene and protein expression of PTPRD in healthy tissues/organs and in patients with EC and GBM. Results: PTPRD exhibits high expression in the occipital lobe, parietal lobe, globus pallidus, ventral thalamus, and white matter, whereas in the human placenta, it is primarily localised around the tertiary villi. PTPRD is significantly upregulated at the mRNA and protein levels in patients with EC and GBM compared to healthy controls. In patients with EC, PTPRD is significantly downregulated with obesity, whilst it is also expressed in the peripheral leukocytes. The EC TMAs revealed abundant PTPRD expression in both low- and high-grade tumours. Asprosin treatment upregulated the expression of PTPRD only in syncytialised placental cells. Conclusions: Our data indicate that PTPRD may have potential as a biomarker for malignancies such as EC and GBM, further implicating asprosin as a potential metabolic regulator in these cancers. Future studies are needed to explore the potential molecular mechanisms/signalling pathways that link PTPRD and asprosin in cancer.

## 1. Introduction

Asprosin is a protein which was first described as an adipokine by Romere and colleagues in 2016 during studies of a rare inherited disorder called neonatal progeroid syndrome (NPS) [1]. Neonates with NPS are characterised by an elevated metabolic rate, low appetite, and insulin resistance in the presence of euglycemia; low subcutaneous body fat; and a markedly low body weight. When studying NPS, Romere et al. discovered a truncated heterozygous mutant form of the fibrillin-1 (FBN1) gene, which encodes the protein profibrillin-1 that is later cleaved to active fibrillin-1 and asprosin by the enzyme furin. As this genetic mutation resulted in lower levels of asprosin, Romere et al. hypothesised that this might be the cause of the altered metabolism in patients with NPS. Since its discovery, an increasing number of publications have made a case for asprosin in regulating metabolic homeostasis and other physiological processes [2]. For example, asprosin is shown to be involved in appetite regulation at the hypothalamic level, as well as in hepatic gluconeogenesis. Moreover, there is also an increasing body of evidence to suggest that asprosin is associated with metabolic disorders and complications during pregnancy, such as gestational diabetes (GDM), and preeclampsia, as well as intra-uterine growth restriction [3,4,5,6]. Additionally, elevated circulating asprosin levels have been noted in women with polycystic ovary syndrome (PCOS), which, together with obesity and diabetes, are all significant risk factors for endometrial cancer [7].

To date, there is a limited number of studies in the literature on the role of asprosin in cancer. Previously published work by our research group has explored the role of asprosin in cancer by studying asprosin and its proposed cognate receptor Olfactory Receptor Family 4 Subfamily M Member 1 (OR4M1) [8]. Indeed, we have shown that the treatment of ovarian cancer cells in vitro with asprosin results in the alteration of pathways associated with cell communication, TGF-β signalling, and cell proliferation [9]. More recently, it was also shown that circulating asprosin levels can act as a potential biomarker in ovarian cancer, distinguishing serous benign, serous borderline, and malignant ovarian cancers [10]. Similarly, the clinical utility of serum asprosin levels was also noted in early pancreatic cancer, whilst a significant increase in asprosin immuno-reactivity was also noted in colorectal adenocarcinoma (i.e., grade 1 vs. grade 2) [11,12].

Moreover, asprosin appears to exert its effect in a tissue- and organ-specific manner, activating a number of receptors, including OR4M1, Toll-Like Receptor 4 (TLR4), and recently, Protein Tyrosine Phosphatase Receptor Type D (PTPRD) [2,13]. The latter is involved in the regulation of various biological processes, including cell growth, differentiation, and oncogenic transformation [14]. Moreover, PTPRD has been involved in regulating neuronal survival and differentiation during brain development via its effects upon cell growth and differentiation by directly dephosphorylating specific protein substrates on tyrosine residues [15]. Thus, PTPRD may act as an intracellular transducer during neuronal development, responding to external stimuli that drive cell proliferation, survival, and differentiation [16]. Furthermore, PTPRD appears to be involved in the regulation of cognitive functions, such as learning and memory, since PTPRD-deficient mice have impaired cognitive functions, including deficits in contextual fear memory and spatial learning [17]. Notably, until recently, PTPRD had no known ligand; however, Mishra et al. demonstrated that PTPRD may be the asprosin receptor associated with its orexigenic effects in the hypothalamus by showing that asprosin functions as a high-affinity PTPRD ligand in hypothalamic agouti-related protein (AgRP) neurons. The genetic ablation of PTPRD resulted in strong losses of appetite and leanness and an inability to respond to the orexigenic effects of asprosin in mice [13]. 

Given the emerging evidence on asprosin and the potential role of PTPRD in regulating cancer cell growth and metastasis, and a recent genome-wide association study (GWAS) meta-analysis indicating that a locus located within PTPRD is associated with endometrial cancer, we investigated its expression in this gynaecological malignancy, which is the fourth most common cancer and the third leading cause of cancer mortality in females worldwide [18,19]. We have used a number of in silico and in vitro approaches, as well as clinical samples from endometrial cancer patients. Furthermore, we have expanded our observations on the expression of PTPRD in glioblastoma multiforme (GBM; a highly heterogeneous type of brain tumour, characterised by rapid cell division and extensive proliferation). As such, our in silico analysis sought to examine the expression patterns and regional and cell-specific distributions of the PTPRD protein within cohorts of patients with and without GBM, and we conducted cellular localisation in vitro. Finally, we also explored the expression of PTPRD in human placental cell lines and placental tissue, since foetal genetic loci close to PTPRD—previously implicated in neurodevelopment—may be of use as biomarkers for environmental exposures during pregnancy, whilst asprosin can act as a biomarker in pregnancy complications associated with maternal obesity (e.g., GDM) [20].

## 2. Materials and Methods

In silico tools were used to determine protein and gene expression of PTPRD.

The Human Protein Atlas (HPA) (https://www.proteinatlas.org/, accessed on 10 May 2023) was used to investigate the mRNA expression of PTPRD [21]. HPA-RNA-seq data were employed to examine the healthy tissue expression of PTPRD across various tissue types measured in normalised transcripts per million (nTPM). This tool was also utilised to obtain data on PTPRD mRNA expression across 17 different cancer types. These data were acquired from The Cancer Genome Atlas (TCGA) database and were measured in number of fragments per kilobase of exon per million (FPKM). To visualise the expression of PTPRD across six brain donors (NM_014325.2), the Allen Brain Atlas (https://portal.brain-map.org/, accessed on 10 May 2023) was incorporated, and these results are presented as heatmaps of log2 expression [22]. The data used in the corresponding figure are publicly available from the Allen Brain Atlas. Furthermore, the UALCAN database (https://ualcan.path.uab.edu/, accessed on 10 May 2023) was used to evaluate the different mRNA expressions of PTPRD among healthy and GBM cohorts (TCGA: https://ualcan.path.uab.edu/analysis.html, accessed on 10 May 2023) [23,24]. The significance of the TPM values used by UALCAN to generate box plots was assessed using a *t*-test PERL script with the CPAN module “Statistics: *t*-Test”. Values of *p* < 0.05 were considered statistically significant. Statistically analysed data via one-way ANOVA to compare disease states (tumour or Healthy) were obtained from GEPIA2 (http://gepia2.cancer-pku.cn/#index, accessed on 5 June 2023) [25]. Genes that met the pre-set thresholds of higher log2FC values and lower q values were considered statistically significant at a *p*-value of < 0.01. STRING (Search Tool for the Retrieval of Interacting Genes/Proteins) was also used to determine protein–protein interactions [26].

Lastly, the single-cell portal was utilised to examine the log2 expression of PTPRD within healthy brain tissues (processed human expression data are available from the Single Cell Portal; https://portals.broadinstitute.org/single_cell/study/SCP381/experiment-4-human-st, accessed on 5 June 2023), ten astrocytoma cases (available from the Single Cell Portal; https://singlecell.broadinstitute.org/single_cell/study/SCP50/single-cell-rna-seq-analysis-of-astrocytoma, accessed on 5 June 2023), and 28 GBM cases (GSE131928), among which there were 8 from a paediatric cohort and 20 from an adult cohort (https://singlecell.broadinstitute.org/single_cell, accessed on 5 June 2023) [27].

### 2.1. Cell Culture

Endometrial cancer cells, Ishikawa, AN3-Ca, RAL95-2, and NOU-1 cells were cultured using a complete media of either Dulbecco’s Modified Eagle’s Medium (DMEM, Gibco, Bleiswijk, The Netherlands), High-Glucose Liquid Medium (Cytiva, Amersham, UK), or RPMI (Cytiva) as per the manufacturers’ instructions and supplemented with 10% Foetal Bovine Serum (FBS, Gibco, Bleiswijk, The Netherlands) and 1% penicillin-streptomycin (Gibco) at 37 °C with 5% CO_2_. BeWo placental cells were cultured using Dulbecco’s Modified Eagle’s Medium (DMEM) Ham’s F12 (Sigma Aldrich D8437, Gillingham, UK) supplemented with 10% Foetal Bovine Serum (Sigma Aldrich F6765) and 1% penicillin-streptomycin (Gibco 15140122) at 37 °C with 5% CO_2_. BeWo cells were stimulated to syncytialise via treatment with forskolin (Sigma Aldrich, F3917), reconstituted in Dimethyl Sulfoxide (DMSO, Sigma Aldrich, D2650). Cells were treated with 20 μM forskolin for 48 h to stimulate syncytialisation. JEG3 placental cells were cultured using Eagle’s minimum essential medium (EBSS) with 2 mM Glutamine, 1% Non-Essential Amino Acids (NEAAs), 1 mM Sodium Pyruvate (NaP), 10% Foetal Bovine Serum, and 1% penicillin-streptomycin (Gibco) at 37 °C with 5% CO_2_. The A172 and U251MG glioblastoma cell lines were purchased from the American Type Culture Collection (ATCC, Manassas, VA, USA). A172 cells were grown in complete DMEM (Gibco, Bleiswijk, The Netherlands) supplemented with 10% FBS (Gibco) and 1% Pen/Strep (Gibco). U251MG cells were cultured in complete minimum essential media (MEM, Gibco, Bleiswijk, The Netherlands) supplemented with 10% FBS and 1% Pen/Strep. Both cell lines were maintained at 37 °C with 5% CO_2_.

### 2.2. Immunofluorescence (IF)

In preparation for immunofluorescence (IF), an 8 mm coverslip was added to each well of a 6-well plate under a laminar flow cabinet. In the same method used for subculture, Ishikawa, AN3Ca, RAL95-2, and NOU-1 cells were resuspended in complete media and incubated for 24 h or until they reached a confluence of approximately 80% on the coverslip. The 6-well plate was then removed from cell culture conditions. Media were aspirated, and cells were washed twice with 1 mL of PBS. Fixation of cells was undertaken using 4% paraformaldehyde (PFA) for five minutes. Repeat washings were undertaken by applying solution away from the coverslip to avoid detachment of cells. No permeabilisation of the cell was undertaken. Blocking was then undertaken using 100 μL of 1% bovine serum albumin (BSA) diluted in PBS in each well. Parafilm was used to prevent dehydration, and the slides were left for one hour at room temperature. Next, 100 μL of the primary antibody solution diluted in 1% BSA in PBS was applied. PTPRD antibody (Biotechne, Abingdon, UK NBP2-49153-25 µL) was trialled at concentrations of 1:200, 1:100, and 1:50. Following incubation with the primary antibody, 1 mL of PBS was then used to wash the coverslips three times for five minutes each. The secondary antibody, anti-Rabbit Alexa Fluor 488 antibody (Merck Millipore, Watford, UK), was added to each well at a concentration of 1:200 and left for one hour in the dark at room temperature. A final three washes with PBS were then undertaken. Coverslips were removed from the six-well plate and allowed to airdry. Glass slides were prepared with 5 μL of mounting medium with DAPI nuclear stain (Vectashield), and cover slides were inverted gently onto the mounting media and left for ten minutes to allow the mounting media to dry. Slides were sealed with clear nail varnish and left to air dry before viewing under a LEICA DM4000 Fluorescent Microscope. All IF analyses were carried out using the LAS-X analysis software (version 3.7.0).

### 2.3. Immunohistochemistry (IHC) of Tissue Microarray 

A paraffin-embedded tissue microarray (AMSBIO) was used for the present IHC work. This consisted of 41 cases of endometrioid adenocarcinoma, 2 serous adenocarcinoma, 2 clear cell adenocarcinoma, and 5 cases of healthy endometrial tissue. The specification sheet containing information about patient age and tumour grade and stage was reviewed prior to purchase (Supplementary Table S1). Tissue samples were collected under Health Insurance Portability and Accountability Act’s (HIPAA) approved protocols and ethical standards. Unless otherwise stated, reagents were purchased from Thermo Fisher Scientific (Waltham, MA, USA). Slides were initially deparaffinised and rehydrated. Following this, the slides were boiled in pre-warmed sodium citrate solution (10 mM sodium citrate in distilled water, 0.05% Tween 20, pH 6.0) to facilitate antigen retrieval. Slides were then washed in 0.025% Triton X in PBS (Thermo Fisher Scientific, Inc.) before being incubated in 3% H_2_O_2_. A further three washes in 0.025% Triton X in PBS followed. The slides were blocked with 5% BSA in PBS, followed by incubation with 200 μL PTPRD antibody (1:50) overnight in a humidity chamber at 4 °C. Parafilm was placed over the slides to prevent it from drying out. Slides were then washed three times in 0.025% Triton X in PBS and incubated with a secondary antibody in 1% rabbit serum (ZytoChem Plus HRP DAB Kit, Zytomed Systems GmbH, Upper Heyford, Oxfordshire, UK) for 1 h. The slides were then washed with 0.025% Triton X in PBS to ensure the removal of unbound secondary antibody. Streptavidin HRP conjugate was then added to the bound secondary antibody, and the slide was incubated for a further 30 min inside the humidity chamber. Slides were washed with PBS before the addition of the DAB stain. These were then counterstained with haematoxylin and washed with 0.1% sodium bicarbonate. Finally, slides were dehydrated before the addition of DPX and coverslips, and then left to dry overnight. Immunoreactivity was analysed using a light microscope (Zeiss GmbH, Cambridge, UK). Results were calculated by three independent reviewers using a percentage score of positive tumour cells, as described previously [28].

### 2.4. RNA Isolation, cDNA Synthesis, and Reverse Transcription Quantitative PCR (RT qPCR)

Total RNA was extracted from cell lysates using the RNeasy Mini Kit (Qiagen, Inc., Manchester, UK). Sample purity was assessed using Nano Drop 2000C (Thermo Fisher Scientific, Inc.). Samples were then reverse-transcribed to cDNA using a cDNA reverse transcription kit (Applied Biosystems, Waltham, MA, USA; Thermo Fisher Scientific, Inc.). RT-qPCR was then undertaken using the QuantStudio 7 Flex Real-Time PCR Machine and SYBR TM Green PCR Master Mix (Applied Biosystems). Primers for PTPRD were designed according to the Harvard Primer Bank (Table 1). YWHAZ was used as house-keeping gene in accordance with the literature for endometrial cancer cells [29]. RQ values were calculated, as previously described, according to the comparative 2^ΔΔCq^ analysis method [30]. 

### 2.5. Imaging Flow Cytometry of Clinical Samples

Venous blood samples were collected from patients with endometrial cancer recruited at the Royal Surrey NHS Foundation Trust, United Kingdom as part of an ongoing clinical study assessing liquid biopsy diagnostic accuracy for EC detection (n = 50). For the blood sample processing, red blood cells (RBCs) were separated from the remaining sample containing white blood cells. Firstly, 1 mL of blood sample was added to 9 mL of RBC lysis buffer. This was inverted 10 times and then incubated at a shaker for 10 min. After that, it was centrifuged at 2500 RPM for 10 min. Next, the supernatant was discarded, and again, 3 mL of RBC lysis buffer was added and mixed with pellet. This was incubated again in a shaker and re-centrifuged. Next, the pellet was re-suspended and fixed in 4% PFA. Then, 1 mL of 5% BSA in PBS was added and left to incubate for an hour, followed by the addition of PTPRD antibody (Biotechne Ltd., Abingdon, UK) dissolved in blocking buffer at 1:300 dilution and 1:100 conjugated CD45/LCA PE-Texas Red^®^ (Life Technologies, Carlsbad, CA, USA), and left to incubate for an hour. The secondary antibody (Merck Millipore, Watford, UK) was added at 1:1000 dilution for samples with PTPRD antibody and left for one-hour incubation. Following this incubation period, the sample was centrifuged at 4000 rpm for 3 min and washed with 0.1% Tween in PBS, followed by a second centrifugation with the same settings. Finally, 100 µL of Accumax cell detachment solution (StemCell Technologies, Cambridge, UK) was added to the pellet alongside 0.5 µL of the nuclear staining DRAQ5 (BioStatus, Loughborough, UK), and it was ready to be run on the Imagestream Mark II (Cytek Biosciences, Bethesda, MD, USA).

### 2.6. Statistical Analysis

Statistical analyses were performed using GraphPad Prism9^®^ software (GraphPad Software, Inc., La Jolla, CA, USA). Error bars in graphs are presented as standard error of the mean (SEM). Mann–Whitney U test and a one-way ANOVA (Analysis of Variance) with Tukey’s multiple-comparison post hoc statistical test were applied to the observed measurements from the data. The method for differential analysis conducted by GEPIA is listed as a one-way ANOVA, where disease state (tumour or Healthy) is used as a variable for calculating differential expression: gene expression against disease state. The expression data are first log2(TPM + 1) transformed for differential analysis, and the log2FC values are defined as the tumour median and healthy median. Genes with higher |log2FC| values and lower q values than the preset thresholds are considered differentially expressed genes. Unless stated otherwise, significance was set at a *p*-value of <0.05.

## 3. Results

### 3.1. PTPRD mRNA Expression across Human Tissues

**Figure 1:**
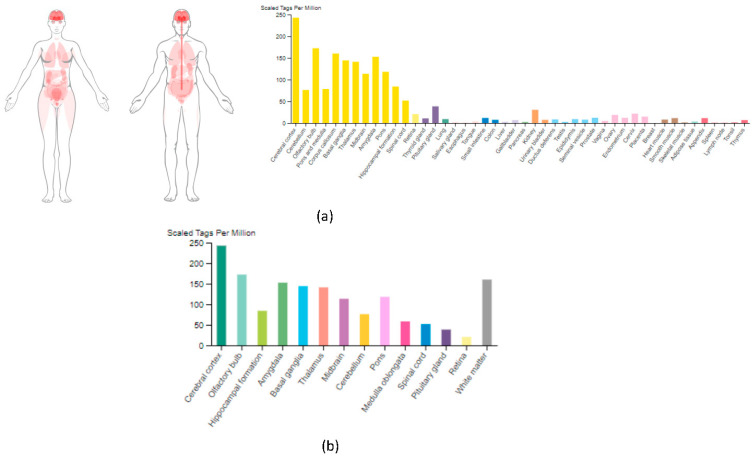
Protein Tyrosine Phosphatase Receptor Type D (PTPRD) mRNA expression examined across healthy human tissues [9,10]. (**a**) The detection of PTPRD mRNA expression within different tissues was performed via the Human Protein Atlas database. Higher expression levels were observed in brain regions, including the cerebral cortex and amygdala, whereas moderate to low expression levels were detected within the pituitary gland, kidney, cervix, and ovary tissues. (**b**) Analysis of *PTPRD* mRNA distribution throughout the brain revealed its highest expression in the cerebral cortex, olfactory bulb, and the white matter regions.

Figure 1 presents the mRNA expression profile of PTPRD in healthy human tissues. In Figure 1a, the highest PTPRD expression, measured in normalised transcripts per million (nTPM), was observed within the brain, particularly in the cerebral cortex (244.6 nTPM). Other tissues presented moderate to low expressions of PTPRD, including the kidney (30.7 nTPM), cervix (21.7 nTPM), and ovary (18.8 nTPM). When examining the brain tissue and its associated regions separately (Figure 1b), high distributions of PTPRD expression were noted across the cerebral cortex (244.6 nTPM), olfactory bulb (173.5 nTPM), and the white matter (161.2 nTPM). Furthermore, a moderately high expression of PTPRD mRNA was shown in the rest of the brain regions (Figure 1b), including the basal ganglia, thalamus, pons, and midbrain (145.3 nTPM, 142.1 nTPM, 118.8 nTPM, and 114.0 nTPM, respectively), followed by the hippocampal formation and cerebellum (84.7 nTPM and 76.7 nTPM, respectively).

### 3.2. PTPRD Regional Distribution within the Brain 

**Figure 2:**
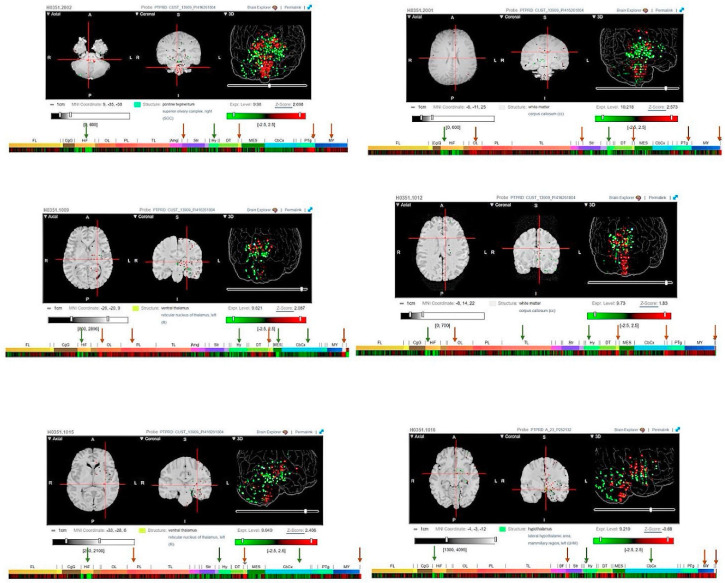
Log2 expression heatmaps generated from microarray experiments conducted on six healthy donors (data obtained from the Allen Brain Atlas). An analysis of the Allen Brain Atlas database indicated that Protein Tyrosine Phosphatase Receptor Type D (PTPRD) exhibits high expression levels in the occipital lobe, parietal lobe, globus pallidus, ventral thalamus, and white matter, and high to moderate expression within the hippocampal formation, hypothalamus, myelencephalon, and cerebral cortex regions of the brain (green arrows—moderate to high expression; orange arrows—predominantly high expression) [31].

The expression of *PTPRD* in various human brain regions of six healthy individuals was evaluated using heatmaps displaying log2 expression values. A remarkably high expression of *PTPRD* was observed within the globus pallidus, ventral thalamus, white matter, pontine tegmentum, and myelencephalon regions of the brain. Figure 2 depicts Magnetic Resonance Imaging (MRI) scans and the corresponding heatmaps from these six healthy adult donors who did not present with any brain pathology prior to death. Notably, *PTPRD* expression was prominently present in the occipital lobe (log2 expression 9.8 ± 2.5), globus pallidus (log2 expression 9.6 ± 2.5), ventral thalamus (log2 expression 10.05 ± 2.5), pontine tegmentum (log2 expression 9.7 ± 2.5), and white matter (log2 expression 10.7 ± 2.5) across all six donors. Additionally, the heatmaps revealed elevated to moderate expression levels of *PTPRD* in the myelencephalon (log2 expression 9.656 ± 2.5), hippocampal formation (log2 expression 7.8 ± 2.5), hypothalamus (log2 expression 7.8 ± 2.5), and cerebral cortex (log2 expression 8.8 ± 2.5) regions of the brain. The cell clusters obtained from healthy brain tissues were used to examine the relative expression of *PTPRD* within astrocytes, endothelial cells, GABAergic neurones, glutamatergic neurones, microglia, oligodendrocytes, and oligodendrocyte precursor cells (OPC) (Supplementary Figure S1).

### 3.3. PTPRD Relative Expression in Endometrial Cancer and GBM 

**Figure 3:**
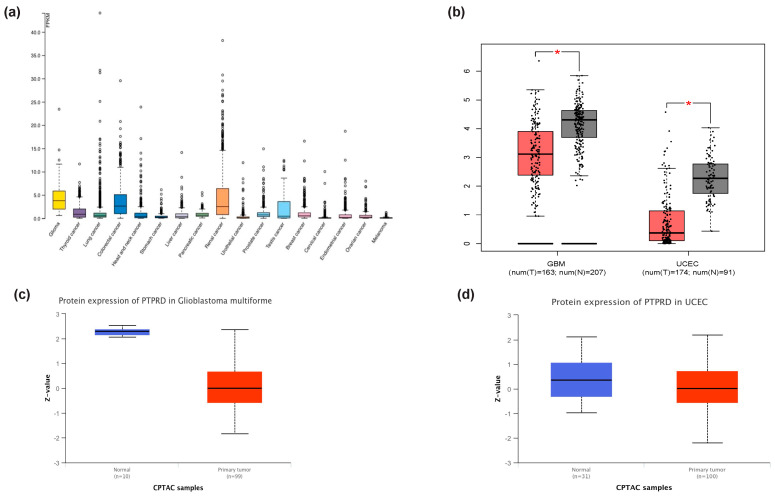
The expression pattern of *Protein Tyrosine Phosphatase Receptor Type D (PTPRD)* in different cancer types. (**a**) Comparison between 17 cancer types revealed moderately low expression of *PTPRD* in gliomas, colorectal, and renal cancers. Significant downregulation of *PTPRD* in glioblastoma multiforme (GBM) and uterine corpus endometrial carcinoma (UCEC) (**b**) tumour samples (red) compared to healthy brain tissue (grey) (* *p* < 0.01, one-way ANOVA) was seen through incorporating GEPIA2. *PTPRD* was similarly downregulated in both GBM (**c**) and UCEC (**d**) tumour samples at the protein level compared to healthy cohorts (* *p* < 0.05). TPM: transcripts per million. CPTAC: Clinical Proteomic Tumor Analysis Consortium.

**Figure 4:**
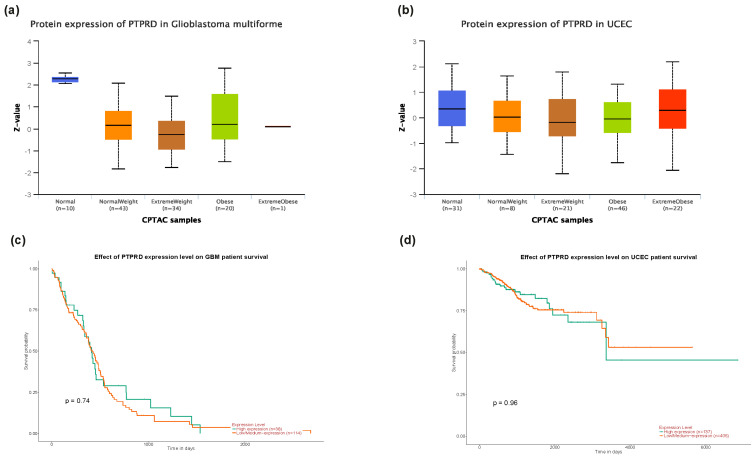
No statistical significance was depicted between the different GBM groups when assessed in terms of their body weights (**a**). *PTPRD* expression was significantly lower in the UCEC cohort with obesity compared to the healthy body weight group (**b**). Difference between the high expression cohort and the low/medium expression cohort was not significant in terms of overall survival for GBM (**c**) and UCEC (**d**), with *p*-values of 0.74 and 0.96, respectively. CPTAC: Clinical Proteomic Tumor Analysis Consortium.

**Figure 5:**
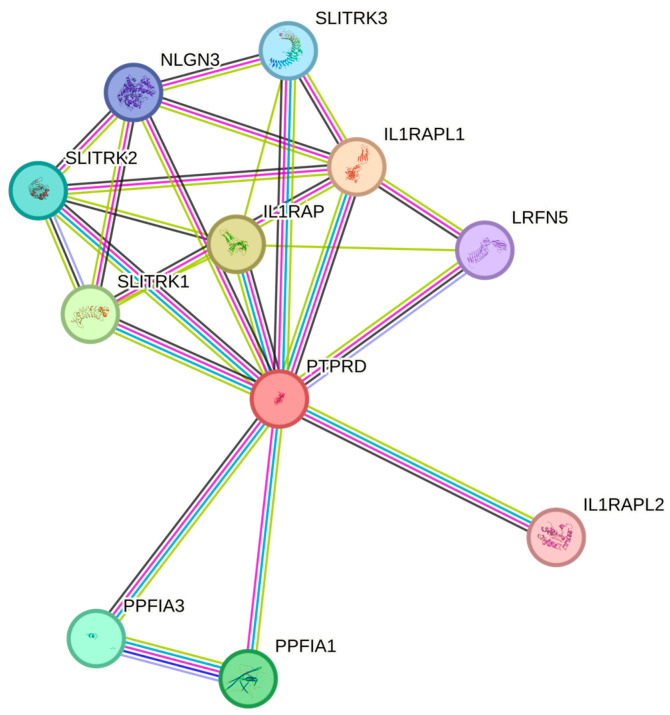
PTPRD can potentially interact with Interleukin-1 receptor accessory protein-like 1 (L1RAPL1), Interleukin-1 receptor accessory protein (IL1RAP), Liprin-alpha-1 (PPFIA1), Liprin-alpha-3 (PPFIA3), SLIT and NTRK-like protein 1 (SLITRK1), SLIT and NTRK-like protein 2 (SLITRK2), SLIT and NTRK-like protein 3 (SLITRK3), Neuroligin-3 (NLGN3), Leucine-rich repeat and fibronectin type-III domain-containing protein 5 (LRFN5), and Interleukin-1 receptor accessory protein like 2 (IL1RAPL2).

RNA-sequencing (RNA-seq) data from the TCGA database was utilised to assess the specificity of PTPRD across various cancer types, as presented in Figure 3a. These RNA-seq data were quantified in terms of the median number of fragments per kilobase of exon per million reads (FPKMs). Among the 17 different cancer types examined, gliomas exhibited the highest median FPKM expression levels for PTPRD, followed by colorectal and renal cancers, with values of 3.8 FPKMs, 2.6 FPKMs, and 2.5 FPKMs, respectively. A subsequent analysis of PTPRD expression in both healthy and cancerous brains (GBM) and endometrial (uterine corpus endometrial carcinoma, UCEC) samples revealed a significant downregulation of the gene in the tumour cohort (Figure 3b), with a statistical significance of *p* < 0.01. This significant gene downregulation was also mirrored at the protein level for both GBM (Figure 3c) and UCEC (Figure 3d); *p* < 0.05. Given the role of asprosin in metabolism, appetite regulation, and obesity, we measured PTPRD expression while using body weight as a factor for both malignancies. For GBM, PTPRD expression was unrelated to obesity (Figure 4a), whereas in UCEC, PTPRD was significantly downregulated in UCEC patients with obesity (Figure 4b). In terms of overall survival, PTPRD did not appear to be a good prognostic biomarker for GBM (Figure 4c) nor UCEC (Figure 4d). A protein–protein interaction network analysis using STRING revealed 11 nodes of interactions. Essentially, PTPRD can potentially interact with Interleukin-1 receptor accessory protein-like 1 (L1RAPL1), Interleukin-1 receptor accessory protein (IL1RAP), Liprin-alpha-1 (PPFIA1), Liprin-alpha-3 (PPFIA3), SLIT and NTRK-like protein 1 (SLITRK1), SLIT and NTRK-like protein 2 (SLITRK2), SLIT and NTRK-like protein 3 (SLITRK3), Neuroligin-3 (NLGN3), Leucine-rich repeat and fibronectin type-III domain-containing protein 5 (LRFN5), and Interleukin-1 receptor accessory protein like 2 (IL1RAPL2) (Figure 5). Based on a functional enrichment of the network, a number of biological processes (Table 2) and molecular functions (Table 3) have been identified. 

### 3.4. PTPRD Expression in Endometrial Cancer

**Figure 6:**
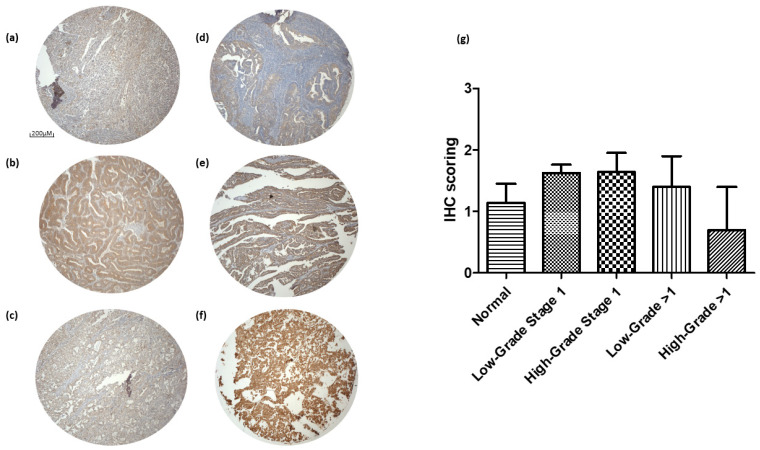
Protein Tyrosine Phosphatase Receptor Type D (PTPRD) expression in of a tissue microarray containing 43 endometrial cancer cores and 5 healthy tissue cores. Healthy (**panel a**), low-grade stage 1 (**panel b**), high-grade stage 1 (**panel c**), low-grade stage >1 (**panel d**), high-grade stage >1 (**panel e**), and positive control adrenal tissues (**panel f**). Immunohistochemical (IHC) scoring ± standard error of the mean (SEM) (**panel g**). Scale bar for images (**a**–**f**): 200 μM.

An IHC analysis of a tissue microarray containing 43 endometrial cancer cores and 5 healthy tissue cores, each representing a different clinical case, was also used to measure PTPRD expression and cellular distribution. PTPRD was aberrantly expressed across all different grades and stages. When compared to the controls, there was no apparent statistical significance in the expression of PTPRD (Figure 6). The mean IHC staining value for PTPRD was 1.24 ± 0.46 in the healthy tissues (n = 5); 1.56 ± 0.13 in the low-grade stage 1 tissues (n = 30); 1.68 ± 0.28 in the high-grade stage 1 tissues (n = 9); 1.36 ± 0.44 in the low-grade stage >1 tissues (n = 2); and 0.68 ± 0.68 in the high-grade stage >1 tissues (n = 2).

**Figure 7:**
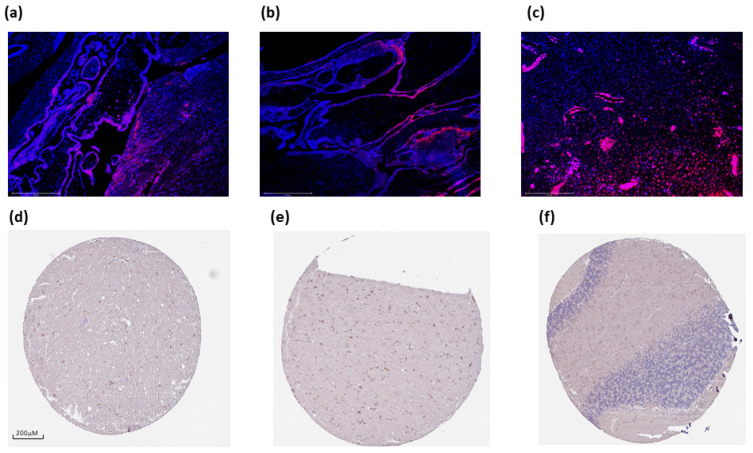
Protein Tyrosine Phosphatase Receptor Type D (PTPRD) expression in the human placenta and brain. PTPRD is widely expressed around syncytiotrophoblasts and cytotrophoblasts (**panels a**–**c**). Moderate expression was also evident in the hippocampus (**panel d**, female, age 52; HPA054829), cerebral cortex (**panel e**, male, age 70; HPA054829), and cerebellum (**panel f**, female, age 54; HPA054829). Scale bar: 275 μM (**a**–**c**), and 200 μM (**d**–**f**).

In addition, we expanded on our observations using the HPA database for brain tissues and placental formalin-fixed paraffin-embedded (FFPE) tissues (n = 3, Figure 7). PTPRD is aberrantly expressed in syncytiotrophoblasts in the tertiary villi of the human placenta (Figure 7a–c). In the brain, there is moderate cytoplasmic/membrane staining of glial cells in the hippocampus (Figure 7d), moderate cytoplasmic/membrane staining of glial cells, but not in the endothelial or neuronal cells in the cerebral cortex (Figure 7e), and weak staining in the cells in the granular area of the cerebellum; no staining was detected in the Purkinje cells (Figure 7f).

**Figure 8:**
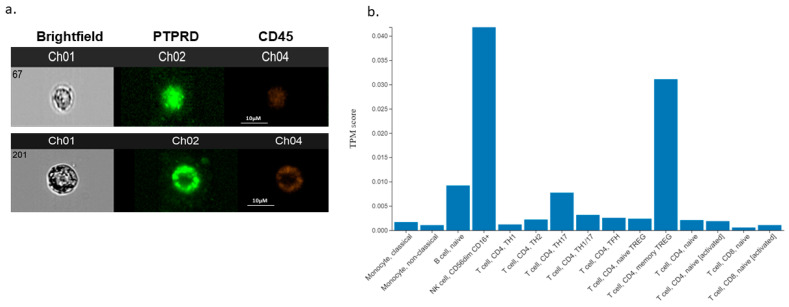
Captured images from blood samples of EC using ImageStream™ (**panel a**). Channel 1 (Ch01) shows the brightfield images of cells, Ch02 represents PTPRD antibody staining, and Ch04 indicates CD45 positive staining as a marker for white blood cells. In silico expression of PTPRD in white blood cells (**panel b**). Scale bar for ImageStream™ images (**panel a**): 10 μM.

We also expanded on the expression of PTPRD in endometrial cancer by measuring its expression in the blood of patients with endometrial cancer. Thus, we demonstrate, for first time, that patients with endometrial cancer express PTPRD in their white blood cells (CD45 positive, Figure 8a). Moreover, the in silico analysis results corroborate these data, since a wide repertoire of white blood cells, including T cells and monocytes, appear to express PTPRD (Figure 8b). 

### 3.5. PTPRD Expression In Vitro 

**Figure 9:**
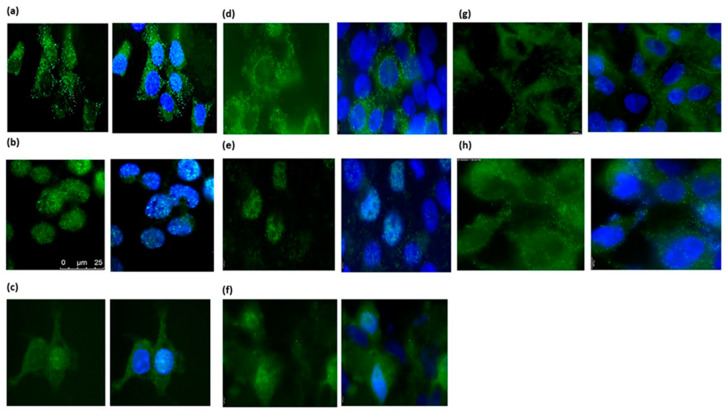
Protein Tyrosine Phosphatase Receptor Type D (PTPRD) expression in Ishikawa (**panel a**), ANC3A (**panel b**), NOU-1 (**panel c**), JEG-3 (**panel d**), BeWo (**panel e**), syncytialised BeWo (**panel f**), U251MG (**panel g**), and A172 (**panel h**) cell lines. Scale bar (**a**–**c**): 25 μM.

We further studied the expression of PTPRD in vitro using different human endometrial (Ishikawa, ANC3A, NOU-1) and placental (JEG-3, BeWo) cell lines. PTPRD exhibited a strong cytoplasmic distribution, although nuclear staining was also evident for ANC3A and BeWo cells. Moreover, the U251MG and A172 GBM cell lines also expressed PTPRD abundantly (Figure 9).

### 3.6. Effect of Asprosin on PTPRD Expression

**Figure 10:**
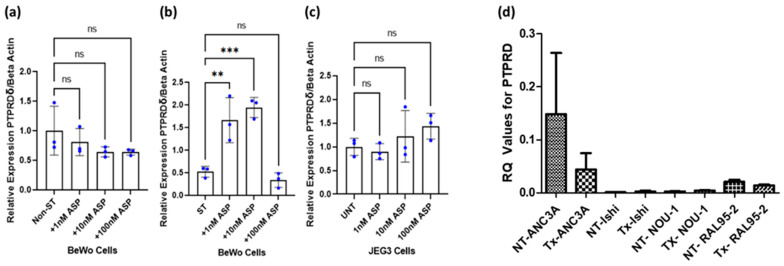
Effect of asprosin (ASP) treatment on *Protein Tyrosine Phosphatase Receptor Type D (PTPRD)* expression on non-syncytialised BeWo cells (Non-ST; **panel a**), syncytialised BeWo cells (ST; **panel b**), JEG-3 cytotrophoblasts (**panel c**) (** *p* < 0.01, *** *p* < 0.0001), and endometrial cancer cell lines (**panel d**); ns: non-significant; UNT: untreated; NT: non-treated, Ishi: Ishikawa. Values ± standard error of the mean (SEM).

Previous studies have shown that asprosin is expressed in cytotrophoblast cells, syncytiotrophoblasts, and in Hofbauer cells in the human placenta villi [6]. Given that PTPRD is expressed in the same cells, it is possible that asprosin acts at the placental level in an autocrine manner. When JEG-3 and BeWo cells were treated with asprosin (1–100 nM), no apparent changes in the expression of PTPRD were noted (Figure 10a,c). However, in syncytialised BeWo (ST) cells, asprosin significantly upregulated the expression of PTPRD at 1 nM and 100 nM (*p* < 0.0001; Figure 8b). Based on HPA studies the human endometrium expresses both FBN1 and furin; therefore, it is possible that this organ can also be a source of asprosin. We therefore expanded on our observations on the effect of asprosin (10 nM) on PTPRD expression in four different endometrial cancer cell lines, namely ANC3A, Ishikawa, NOU-1, and RAL95-2. There was no significant effect of asprosin on the expression of PTPRD in any of the cell lines tested (Figure 10d).

## 4. Discussion

In the present paper, we provide a detailed map of the gene and protein expression of PTPRD in health and disease (i.e., in EC and GBM cancers) using a combination of in silico, in vitro, and clinical sample data. As aforementioned, PTPRD was identified as a potential orexigenic receptor for asprosin in hypothalamic AgRP neurons, with the genetic ablation of PTPRD in mice leading to a loss of appetite, resistance to diet-induced obesity, and a lack of response to asprosin [13]. Further in vivo experiments using a soluble ligand-binding domain of PTPRD corroborated these findings, including the suppression of blood glucose levels via the inhibition of the gluconeogenic hormone asprosin. Notably, this is the third potential receptor for asprosin, since both OR4M1 and TLR4 have been shown to mediate asprosin effects [32]. Our group has shown that OR4M1 is upregulated in ovarian cancer, particularly at early stages (I and II) compared to late stages (III and IV) [8]. Similarly, TLR-4 appears to have a prognostic role in ovarian cancer progression as well [33]. Interestingly, the risk factors for ovarian cancer include insulin resistance, diabetes, and obesity, which are also associated with dysregulated levels of asprosin [34]. Using an artificial neural network algorithm, we have also shown that obesity is by far the biggest risk factor for endometrial cancer [7]. In this study, no apparent changes in protein expression were detected between the early and late stages for PTPRD. These data mirror the in silico gene expression findings (Supplementary Figure S2) and suggest that there is a threshold effect in the primary tumour, and hence, more discrimination between benign and malignant tissue. For example, JUN proto-oncogene, which has a biomarker potential for EC, is only differentially expressed between healthy and tumour samples, but not at different stages [35]. A STRING motif analysis revealed potential protein–protein interactions of PTPRD with the IL-1 receptor. Daley-Brown et al. have shown that there is a cross-talk between the Notch and IL-1 signalling pathways in EC, and that it is associated with invasiveness and chemoresistance [36].

Data from the HPA revealed that PTPRD is predominantly expressed in the human brain (e.g., white matter, cerebral cortex, and olfactory bulb regions). Further analyses using the Allen Brain Atlas (healthy tissues) supported the findings of high PTPRD expression in the white matter, occipital lobe, ventral thalamus, hippocampal formation, hypothalamus, and cerebral cortex, corroborating previous studies [37,38]. As we have shown, the highest PTPRD expression was observed in the cerebral cortex, implying that brain regions exhibiting high levels of PTPRD may be more susceptible to GBM development. Although it is challenging to provide a concise summary of these complex expression patterns, this highlights the importance of dopamine and acetylcholine in functions related to memory and motor control, involving interactions between subcortical areas, the cortex, and the hippocampus. As such, the differential expression PTPRD within these brain regions may serve as a mediator for these circuits.

Moreover, PTPRD is significantly downregulated in endometrial cancer and GBM when compared to a healthy control group, both at the gene and protein levels. This downregulation can potentially compromise the signalling pathways implicated in cell proliferation. For example, in the p16Ink4a knockout RCAS PDGFB/Nestin-tvA glioma mouse model, the downregulation of PTPRD promoted cell proliferation, whereas restoring PTPRD expression in GBM cells resulted in the suppression of cell growth and the induction of apoptosis [39,40]. In gastric cancers, the loss of PTPRD induced CXCL8 and promoted angiogenic and metastatic events via the STAT3 and ERK signalling pathways [41]. PTPRD has also been shown to be involved in colon cancer cell migration via a β-catenin/TCF/CD44 signalling pathway, whereas in lung cancer, PTPRD appears to act as a tumour suppressor gene [42,43]. 

Similarly, PTPRD acts via a STAT3 pathway that is activated in endometrial cancer [44]. Notably, PTPRD is mutated in 11.14% of endometrial samples [45]. In a GWAS meta-analysis, 13 loci were associated with endometrial cancer and endometriosis, with one particular locus located within the PTPRD gene [18]. Here, we have also shown that, although the expression is not influenced by the grade or stage of endometrial cancer, PTPRD was significantly downregulated in patients with endometrial cancer with obesity when compared to healthy-weight controls. Of note, we have shown that a body mass index (BMI) of 25 kg/m^2^ or over increased the risk for endometrial cancer development by 2.0%, whereas a BMI of 30 kg/m^2^ or over increased this risk by 5.2%, and a BMI of 40 kg/m^2^ or over led to an increase of 6.9% [7]. Interestingly, PTPRD lead to the inhibition of adipogenesis when it was over-expressed in 3T3-L1 pre-adipocytes [46]. Collectively, these data point towards a central role of PTPRD not only as a potential tumour suppressor gene, but also as an orexigenic mediator in endometrial cancer. However, obesity does not appear to influence the expression status of PTPRD in GBM patients, and the levels of expression amongst these patients are not of a clinical utility as a prognostic biomarker.

Furthermore, GWAS studies have also implicated PTPRD in other pathologies, including GDM, spontaneous preterm birth, and in foetal genetic loci, contributing to levels of organohalogens [20,47,48,49]. The latter is of importance, since foetal genes expressed during placentation can play a key role modulating the foetal–placental transfer of these synthetic chemicals, which are accumulative in nature and can exert long-term health effects [50]. In this study, PTPRD was expressed in all studied placental cell lines, including the hormonally active syncytialised BeWo cells, which resemble the tertiary villi. Interestingly, in BeWo cells (cytotrophoblasts), as well as in NOU-1 (a poorly differentiated endometrial adenocarcinoma cell line), PTPRD was also localised in the nucleus. To the best of our knowledge, this is the first time that such cellular distribution has been reported for PTPRD. Using different nuclear localisation signal (NLS) predictors, we found that PTPRD contains NLS signals (position 1288 and amino acid sequence: LLYKRKRAES; position 1175 and amino acid sequence RKR), so it is possible that the protein can translocate (Supplementary Figure S3) [51,52,53]. 

Given that asprosin is produced in the human placenta and endometrium, we assessed its effects of PTPRD expression in vitro. Asprosin induced a significant upregulation of PTPRD only in syncytialised BeWo cells, but not in placental trophoblasts. This suggests that asprosin may act not only in an autocrine manner, but also in tissue- and cell-specific manners. Indeed, this might be the case with the study when changes were seen in syncytiotrophoblasts but not in cytotrophoblasts in vitro. Cytotrophoblast cells subserve as precursors for syncytiotrophoblasts as well as extravillous trophoblasts [54]. These cell types have distinct roles, with the syncytiotrophoblasts mediating the endocrine functions of the human placenta [55]. Moreover, there are morphological changes as well: during the fusigenic process, trophoblasts become amorphous, multinucleated giant cells surrounded by one cell membrane [56,57]. It has also been suggested that different epigenetic states exist between syncytiotrophoblast and cytotrophoblast nuclei [58]. Of note, Xu et al. have shown that PTPRD can be reduced through epigenetic regulation [59]. It is possible, therefore, that when cells are in an “endocrine-active” state, they might respond differently to asprosin. In endometrial cell lines, with the exception of ANC3A, it is difficult to draw conclusions, as PTPRD gene expression appears low in Ishikawa, NOU-1, and RAL95-2. It is not unusual for endometrial cell lines to differentially express the same gene. For example, the transcription factor zinc finger E-box-binding homeobox 1 (ZEB1) is highly expressed in ANC3A cells but not in Ishikawa cells [60]. In ANC3A cells, asprosin also downregulated PTPRD, albeit the effect was not significant. Further experimental replicates are needed to establish whether this trend is actual. 

We acknowledge that our study has a number of limitations, since the role of PTPRD in vitro could have been further explored using siRNA or CRISPR-Cas9 followed by multi-omics readouts. For example, previous studies in gastric cells have shown that PTPRD can be successfully silenced using siRNA, affecting subsequent cell proliferation [61,62]. In terms of multi-omics or integrative omics, studies can include changes in the genome, transcriptome, proteome, epigenome, secretome, and metabolome. Moreover, a Principal Component Analysis (PCA) could have provided a better statistical outcome of the protein expression analysis. A larger sample of tissue microarrays will be needed to perform such an analysis. Finally, the asprosin levels could have been measured in both patients with GBM and endometrial cancer and compared to the healthy controls; however, due to ethical restrictions, we were not able to collect plasma samples for such measurements. In terms of liquid biopsies, future studies are needed to confirm whether circulating tumour cells also express PTPRD, and if its expression is altered during treatments. Finally, the methylation of PTPRD should also be studied, since there is evidence that this gene can be hypomethylated in leukocytes of patients with type 2 diabetes [46]. 

## 5. Conclusions

In the present study, we provide novel evidence of potential asprosin-initiated metabolic processes via PTPRD in health and disease. Indeed, our present findings indicate that PTPRD may have potential as a biomarker for certain malignancies (EC and GBM). Future studies are needed to further explore the potential molecular mechanisms/signalling pathways that may link PTPRD and asprosin in cancer.

## Figures and Tables

**Table 1:** List of primers used for qPCR.

Gene	Forward Primer Sequence	Reverse Primer Sequence
YWHAZ	AGACGGAAGGTGCTGAGAAA	GAAGCATTGGGGATCAAGAA
PTPRD	CAGGCGGAAGCGTTAATATCA	TTGGCATATCATCTTCAGGTGTC

**Table 2:** Biological process enrichment analysis.

GO Term	Description
GO:0099545	Trans-synaptic signalling by trans-synaptic complex
GO:0099537	Trans-synaptic signalling
GO:0099560	Synaptic membrane adhesion
GO:0050808	Synapse organisation
GO:0007416	Synapse assembly
GO:0051963	Regulation of synapse assembly
GO:1905606	Regulation of presynaptic assembly
GO:0099151	Regulation of postsynaptic density assembly
GO:0099175	Regulation of postsynaptic organisation
GO:0051128	Regulation of cellular component organisation
GO:0044087	Regulation of cellular component biogenesis
GO:0065008	Regulation of biological quality
GO:0097105	Presynaptic membrane assembly
GO:0099172	Presynaptic organisation
GO:0051965	Positive regulation of synapse assembly
GO:0030182	Neuron differentiation
GO:0098742	Cell–cell adhesion via plasma membrane adhesion molecules
GO:0098609	Cell–cell adhesion
GO:0007155	Cell adhesion

**Table 3:** Molecular function enrichment analysis.

GO Term	Description
GO:0061809	NAD + nucleotidase, cyclic ADP-ribose generation
GO:0050135	NAD(P) + nucleosidase activity
GO:0004908	Interleukin-1 receptor activity

## Data Availability

Data will be available upon reasonable request.

## Supplementary Materials

The following supporting information can be downloaded at https://www.mdpi.com/article/10.3390/cancers16030582/s1, Figure S1: PTPRD gene expression within single-cell clusters; Figure S2: Nuclear localisation signal (NLS) prediction; Figure S3: Nuclear localisation signal (NLS) prediction (a,b). Alphafold-generated structure prediction of PTPRD (c). Table S1: Information about the tissue microarray.
