# Supplementary material for: In Silico and In Vitro Mapping of Receptor-Type Protein Tyrosine Phosphatase Receptor Type D in Health and Disease: Implications for Asprosin Signalling in Endometrial Cancer and Neuroblastoma
Source: Cancers (Basel). 2024 Jan 30;16(3):582. doi: 10.3390/cancers16030582 (PMC10854520; doi:10.3390/cancers16030582)
Supplement: Supplementary file 1 [file cancers-16-00582-s001.zip › cancers-2824708-supplementary.pdf]

cancers

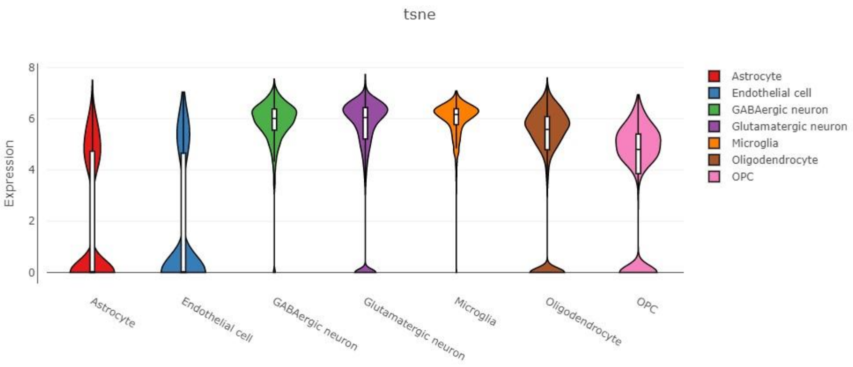

(a)

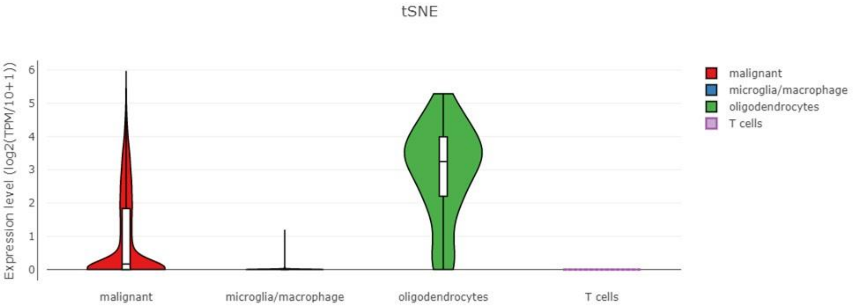

(b)

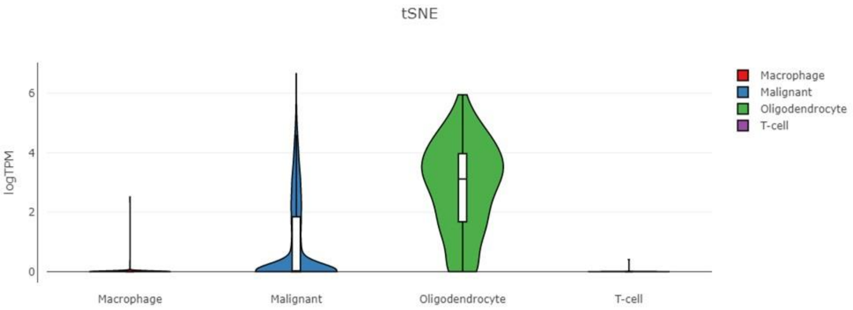

(c)

Supplementary Figure S1. Violin plots of relative *Protein Tyrosine Phosphatase Receptor Type D (PTPRD)* expression within normal and cancerous brain cell clusters. **(a)** All cell types obtained from normal brain cell clusters exhibited *PTPRD* relative gene expression of nearly 8 log2TPM(10+1)**. (b)** Cell clusters obtained from astrocytoma samples revealed lower expression of *PTPRD* in comparison to normal brain cell clusters. The relative expression within malignant cells and oligodendrocytes was 5.9 log2TPM(10+1) and 5.2 log2TPM(10+1), respectively, followed by microglia and T-cells where the levels of *PTPRD* were extremely low or absent**. (c)** IDH-wild type glioblastoma multiforme (GBM) cell cluster-tSNE violin plots showed similar *PTPRD* expression patterns to astrocytoma cell clusters**.**

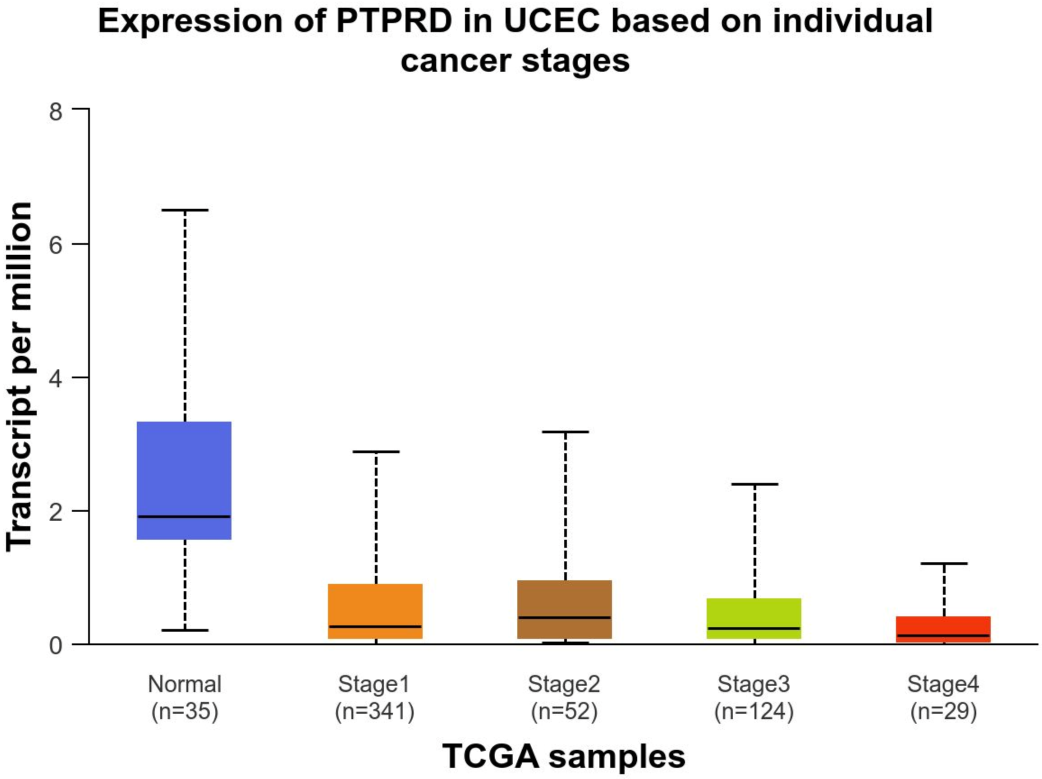

Supplementary Figure S2. Expression of PTPRD in uterine corpus endometrial carcinoma (UCEC), based on differen cancer stages.

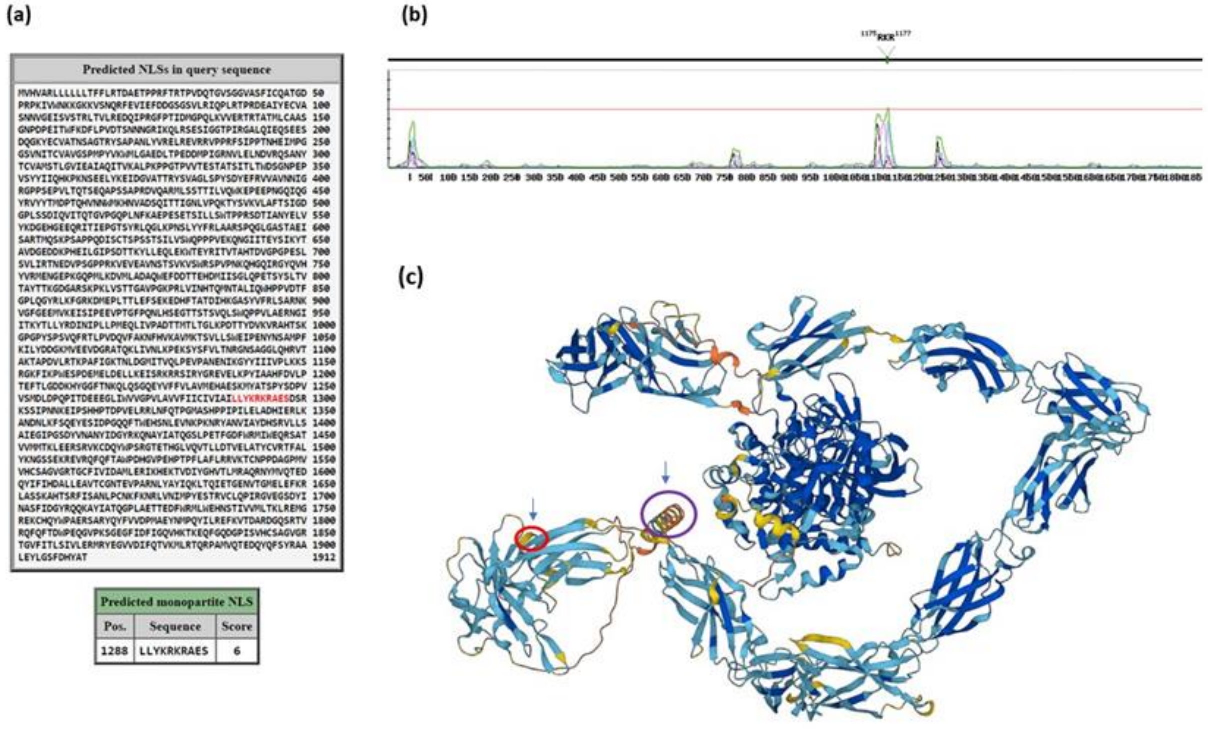

Supplementary Figure S3. Nuclear localisation signal (NLS) prediction (**a** and **b**). Alphafold-generated structure prediction of PTPRD (**c**). Red circle/arrow indicates the RKR NLS, and purple circle/arrow indicates the LLYKRKRAES NLS.

Supplementary Table S1. Information of the tissue microarray. F: female, T: primary tumour site and size, N: regional lymph node involvement; M: presence or otherwise of distant metastatic spread.

Pos.	No.	Age	Sex	Organ/Anatomic Site	Pathology diagnosis	TNM	Grade	Stage	Type
A1	1	40	F	Uterus	Endometrioid adenocarcinoma	T1bN0M0	1	IB	malignant
A2	2	62	F	Uterus	Endometrioid adenocarcinoma	T1N0M0	1	I	malignant
A3	3	63	F	Uterus	Endometrioid adenocarcinoma	T1bN0M0	1	IB	malignant
A4	4	58	F	Uterus	Endometrioid adenocarcinoma	T1cN0M0	1	IC	malignant
A5	5	40	F	Uterus	Endometrioid adenocarcinoma (sparse)	T1bN0M0	1	IB	malignant
A6	6	51	F	Uterus	Endometrioid adenocarcinoma	T1bN0M0	1	IB	malignant
A7	7	57	F	Uterus	Endometrioid adenocarcinoma	T1bN0M0	1	IB	malignant
A8	8	52	F	Uterus	Endometrioid adenocarcinoma	T1cN0M0	1	IC	malignant
A9	9	45	F	Uterus	Endometrioid adenocarcinoma	T1aN0M0	1	IA	malignant
A10	10	51	F	Uterus	Endometrioid adenocarcinoma	T1bN0M0	1	IB	malignant
B1	11	68	F	Uterus	Endometrioid adenocarcinoma	T1cN0M0	1	IC	malignant
B2	12	62	F	Uterus	Endometrioid adenocarcinoma	T1bN0M0	1	IB	malignant
B3	13	59	F	Uterus	Endometrioid adenocarcinoma	T1bN0M0	1	IB	malignant
B4	14	51	F	Uterus	Endometrioid adenocarcinoma	T1bN0M0	1	IB	malignant
B5	15	62	F	Uterus	Endometrioid adenocarcinoma	T1cN1M0	1	IIIC	malignant
B6	16	56	F	Uterus	Endometrioid adenocarcinoma	T1bN0M0	1	IB	malignant
B7	17	62	F	Uterus	Endometrioid adenocarcinoma	T1bN0M0	1	IB	malignant
B8	18	50	F	Uterus	Endometrioid adenocarcinoma	T1bN0M0	1	IB	malignant

B9	19	60	F	Uterus	Endometrioid adenocarcinoma	T1cN0M0	1	IC	malignant
B10	20	51	F	Uterus	Endometrioid adenocarcinoma	T1bN0M0	1--2	IB	malignant
C1	21	60	F	Uterus	Endometrioid adenocarcinoma	T1bN0M0	1--2	IB	malignant
C2	22	55	F	Uterus	Endometrioid adenocarcinoma	T1cN0M0	1--2	IC	malignant
C3	23	61	F	Uterus	Endometrioid adenocarcinoma	T1cN0M0	1	IC	malignant
C4	24	68	F	Uterus	Endometrioid adenocarcinoma	T1cN0M0	1	IC	malignant
C5	25	60	F	Uterus	Endometrioid adenocarcinoma	T1bN0M0	1	IB	malignant
C6	26	50	F	Uterus	Endometrioid adenocarcinoma (smooth muscle tissue)	T1bN0M0	-	IB	malignant
C7	27	52	F	Uterus	Endometrioid adenocarcinoma	T1bN0M0	2	IB	malignant
C8	28	67	F	Uterus	Endometrioid adenocarcinoma	T1cN0M0	1	IC	malignant
C9	29	72	F	Uterus	Endometrioid adenocarcinoma	T1bN0M0	1	IB	malignant
C10	30	55	F	Uterus	Endometrioid adenocarcinoma	T1bN0M0	1	IB	malignant
D1	31	57	F	Uterus	Endometrioid adenocarcinoma	T1bN0M0	2	IB	malignant
D2	32	58	F	Uterus	Endometrioid adenocarcinoma	T1bN0M0	3	IB	malignant
D3	33	52	F	Uterus	Endometrioid adenocarcinoma (smooth muscle tissue)	T1bN0M0	-	IB	malignant
D4	34	53	F	Uterus	Endometrioid adenocarcinoma	T1cN0M0	3	IC	malignant
D5	35	53	F	Uterus	Endometrioid adenocarcinoma	T1cN0M0	3	IC	malignant
D6	36	47	F	Uterus	Endometrioid adenocarcinoma	T1cN0M0	3	IC	malignant
D7	37	62	F	Uterus	Endometrioid adenocarcinoma	T1cN0M0	3	IC	malignant

D8	38	65	F	Uterus	Endometrioid adenocarcinoma with squamous metaplasia	T1bN0M0	3	IB	malignant
D9	39	54	F	Uterus	Endometrioid adenocarcinoma	T1cN0M0	3	IC	malignant
D10	40	56	F	Uterus	Endometrioid adenocarcinoma (chronic inflammion of smooth muscle)	T2aN0M0	-	IIA	malignant
E1	41	48	F	Uterus	Endometrioid adenocarcinoma	T3N0M0	3	III	malignant
E2	42	49	M	Uterus	Serous adenocarcinoma	T1bN0M0	2--3	IB	malignant
E3	43	60	F	Uterus	Serous adenocarcinoma	T3aN0M0	1	IIIA	malignant
E4	44	72	F	Uterus	Clear cell adenocarcinoma	T1cN0M0	2	IC	malignant
E5	45	50	F	Uterus	Clear cell adenocarcinoma	T2bN0M0	2	IIB	malignant
E6	46	21	F	Uterus	Normal endometrium tissue	-	-	-	normal
E7	47	41	F	Uterus	Normal endometrium tissue	-	-	-	normal
E8	48	18	F	Uterus	Normal endometrium tissue	-	-	-	normal
E9	49	21	F	Uterus	Normal endometrium tissue	-	-	-	normal
E10	50	21	F	Uterus	Normal endometrium tissue (smooth muscle and blood vessel)	-	-	-	normal
	0	42	M	Adrenal gland	Pheochromocytoma (tissue marker)		-		Malignant
